# Predictive Factors of 2-Year Postoperative Outcomes in Patients with Spontaneous Cerebellar Hemorrhage

**DOI:** 10.3390/jcm8060818

**Published:** 2019-06-08

**Authors:** Tsung-Han Lee, Yu-Hua Huang, Tsung-Ming Su, Chih-Feng Chen, Cheng-Hsien Lu, Hsiang-Lin Lee, Hui-Ping Tsai, Wen-Wei Sung, Aij-Lie Kwan

**Affiliations:** 1 Division of Neurosurgery, Department of Surgery, Kaohsiung Chang Gung Memorial Hospital and Chang Gung University College of Medicine, Kaohsiung 83301, Taiwan; tsunghan927@gmail.com (T.-H.L.); newlupin2001@yahoo.com.tw (Y.-H.H.); tsungming4516@gmail.com (T.-M.S.); n3518s@yahoo.com.tw (H.-P.T.); 2 Graduate Institute of Medicine, College of Medicine, Kaohsiung Medical University, Kaohsiung 80708, Taiwan; 3 Department of Radiology, China Medical University Hospital, Taichung 40447, Taiwan; xfroggykimo@yahoo.com.tw; 4 Department of Neurology, Kaohsiung Chang Gung Memorial Hospital and Chang Gung University College of Medicine, Kaohsiung 83301, Taiwan; chlu99@ms44.url.com.tw; 5 Department of Surgery, Chung Shan Medical University Hospital, Taichung 40201, Taiwan; s31079@gmail.com; 6 Institute of Medicine, Chung Shan Medical University, Taichung 40201, Taiwan; 7 School of Medicine, Chung Shan Medical University, Taichung 40201, Taiwan; 8 Department of Urology, Chung Shan Medical University Hospital, Taichung 40201, Taiwan; 9 Department of Neurosurgery, Kaohsiung Medical University Hospital, Kaohsiung 80756, Taiwan

**Keywords:** spontaneous cerebellar hemorrhage, Glasgow coma scale, Glasgow outcome scale, National Institutes of Health Stroke Scale, postoperative outcome

## Abstract

Spontaneous cerebellar hemorrhage (SCH) is associated with high patient mortality and morbidity, but the clinical and radiographic predictors of the postoperative outcome have not been widely addressed in the literature. The purpose of this study was to define the prognostic factors for the two-year postoperative outcome in patients with SCH. We conducted a retrospective study of 48 consecutive patients with SCH who underwent neurosurgical intervention. Correlation analysis was performed to examine the possible link between clinical and radiographic parameters, and the National Institutes of Health Stroke Scale (NIHSS) score at each patient’s discharge and the two-year postoperative outcome as defined according to the Glasgow outcome scale (GOS). A total of 48 patients with SCH underwent neurological surgery, which included suboccipital craniectomy and/or external ventricular drainage (EVD). The mean patient age was 63 years. Nine patients underwent suboccipital craniectomy only; 38 underwent both suboccipital craniectomy and EVD. The overall mortality rate was 35.4%. Fourteen patients (29.2%) had good outcomes. A good outcome on the GOS at 2 years after surgical treatment of SCH was associated with the NIHSS score at discharge. An increase of one point in a patient’s NIHSS score at discharge following neurological surgery will increase the probability of a poor two-year postoperative outcome by 28.5%.

## 1. Introduction

Spontaneous intracerebral hemorrhage accounts for 10–15% of all strokes and has a high mortality rate of 19% within the first month [1]. It is the least common type of intracranial hemorrhage, representing 5–10% of all intracerebral hemorrhages, and it is associated with high mortality rates of 20% to 75%, irrespective of treatment [2,3]. Cerebellar hemorrhage is most commonly caused by hypertension [4], but other etiologies may include vascular malformations, aneurysms, coagulopathy, and tumor bleeding [5]. The clinical presentations include an acute onset of dizziness, headache, vomiting, and balance impairment. Some patients that are in good neurological condition can be managed with conservative treatments [5]; however, patients with cerebellar hemorrhage who show neurological deterioration or brainstem compression and/or hydrocephalus from ventricular obstruction are strongly advised to undergo surgical removal of the hemorrhage as soon as possible. Initial treatment of these patients with ventricular drainage, rather than surgical evacuation, is not recommended [6]. 

Currently, the overall surgical mortality rates for cerebral hemorrhages remain as high as 20–50% [3]. In the present study, we retrospectively analyzed data from 48 consecutive patients with a spontaneous cerebellar hemorrhage (SCH) who underwent neurosurgical intervention in our department. The aim of this study was to define the prognostic factors for the two-year postoperative outcome in patients with an SCH.

## 2. Methods

### 2.1. Patients and Study Design

Forty-eight patients (27 men and 21 women; age range: 43–84 years; mean age: 63 years) with an SCH were admitted to our department between 2007 and 2011 and underwent neurological intervention. The protocol was approved by the Institutional Review Board of Chang Gung Medical Foundation (IRB No.: 201600354B0). On admission, each patient’s initial neurological state was scored using the Glasgow Coma Scale (GCS). The data were acquired by a retrospective analysis of the patients’ chart records. The medical records were retrospectively reviewed using the pre-existing standardized evaluation forms, as well as by the brain computed tomography (CT) scan findings for patients admitted with a first-time SCH to the Departments of Neurology and Neurosurgery at the Kaohsiung Chang Gung Memorial Hospital from January 2007 to December 2011. This is a 2718-bed acute-care teaching hospital that provides both primary and tertiary referral care. The hospital’s Institutional Review Committees on Human Research approved the study protocol. The patient evaluations included their demographic data, characteristics, systemic underlying diseases, and other associated features, as well as their neurosurgical interventions. The GCS scores were determined by neurosurgeons or neurologists. The National Institutes of Health Stroke Scale (NIHSS) scores at discharge were determined by the neurologist (Cheng-Hsien Lu). Each patient underwent a brain CT scan upon arrival at the emergency room, as well as a follow-up scan after surgery. Emergency brain CT scans were performed if any of the patients showed clinical deterioration, including the acute onset of focal neurologic deficits and a progressively disturbed consciousness.

The following radiographic parameters were collected, based on a pre-operative CT scan, and were secondarily evaluated by a neuroradiologist (Chih-Feng Chen): The location and maximal diameter of the cerebellar hematoma, the absence or presence of hydrocephalus, any compression of the fourth ventricle, the absence or presence of an intraventricular hematoma, any radiographic signs of brain stem compression, and the absence or presence of a tight posterior fossa. Patients were excluded from the analysis if they: (1) suffered from a non-spontaneous cerebellar hemorrhage, such as a traumatic cerebellar hemorrhage; (2) suffered from a supratentorial, brainstem, or subarachnoid hemorrhage or had an arteriovenous vascular malformation, subdural hematoma, hemorrhagic infarct, or an inflammatory vascular disease; (3) had a spontaneous cerebellar hemorrhage due to a primary or metastatic brain tumor; or (4) had a pre-existing neurological condition with various neurological deficits (e.g., stroke, head trauma, or hypoxic encephalopathy). 

Hydrocephalus was determined retrospectively as a dilated temporal horn of the ventricle without obvious brain atrophy and/or an Evan’s ratio of >0.3 (the ratio of the ventricular width of the bilateral frontal horn to the maximum bi-parietal diameter) on the initial CT scan. Rebleeding was defined as a sudden clinical deterioration accompanied by new or increased bleeding revealed by the brain CT scan [7]. The cerebellar hematoma volume (cc or cm^3^) was obtained by the “ABC/2” formula for a spherical or ellipsoid intracerebral hemorrhage (ICH), where A is the maximum ICH diameter estimated visually, B is the maximum ICH diameter perpendicular to A, and C is the total number of CT slices with the ICH seen in the vertical plane multiplied by the CT slice thickness (typically 5 mm or 0.5 cm). The A, B, and C values are then multiplied together and divided by 2 to give the final volume [8]. A “tight posterior fossa” was defined as an effacement of the basal cisterns of the posterior fossa and an enlargement of the third and lateral ventricles, including the temporal horns, and, occasionally, as an effacement of the fourth ventricle [9]. The patients were defined as diabetic if diabetes mellitus had been previously diagnosed or if they had a fasting glucose level of >7 mmol/L [10]. Hypertension was defined by referring to the patient’s pre-admission history and medical records, and a systolic blood pressure >140 mmHg or diastolic blood pressure >90 mmHg [11]. The evaluations of the 2-year postoperative outcomes were based on the Glasgow Outcome Scale (GOS), which assigns the scores as follows: Grade 1: Death; Grade 2: Vegetative state; Grade 3: Severe disability; Grade 4: Moderate disability; and Grade 5: Good recovery. A follow-up was conducted primarily in the outpatient department and was terminated either by death or by the end of the study (December 2013). For the analysis of the outcomes, good recovery and moderate disability were considered a good outcome, while a GOS score of <4 was considered a poor outcome.

### 2.2. Statistical Analysis

Descriptive statistics were presented as either frequencies (percentages), or as a mean and standard deviation (SD). Separate statistical analyses were conducted to compare patients with good outcomes to those with poor outcomes. A standard level of significance (*p* < 0.05) was used. First, baseline clinical data, including sex and clinical manifestations between the two patient groups, were analyzed by the chi-square test, or by Fisher’s exact test where appropriate. The Mann–Whitney test was used to analyze age, GCS score on admission, pre-operative GCS score, NIHSS score at discharge, and hematoma volume and diameter between the two patient groups. The univariate data with *p* < 0.05 were entered into the multivariate model. The multivariate logistic regression sequentially eliminated factors that did not independently contribute to the postoperative outcome. We examined the effect of each independent risk factor on the postoperative outcome. All statistical analyses were conducted using SPSS version 20.0 (IBM SPSS Statistics).

## 3. Results

### 3.1. Baseline Characteristics

**Figure 1:**
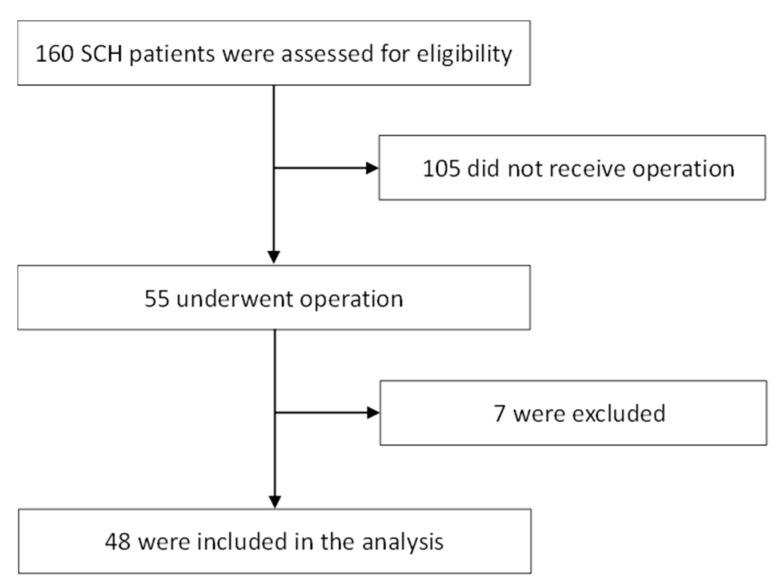
Study flow diagram of this study with 160 patients originally identified with spontaneous cerebellar hemorrhage (SCH). Patients included those with tumor bleeding (*n* = 2) and hemorrhagic infarct (*n* = 2), while those referred from other hospitals after diagnosis and initial treatment (*n* = 3) were excluded.

The study flow diagram shown in Figure 1 describes the 160 patients originally identified and the 112 patients excluded due to either nonsurgical or other conditions. The 48 enrolled patients included 27 men (mean age: 61 years; age range: 43–84 years) and 21 women (mean age: 67 years; age range: 51–80 years). All patients had a history of hypertension. No patient had a systemic bleeding tendency or was taking any anticoagulant or antiplatelet agents. The GCS scores on admission were >8 for 26 patients (54%) and ≤8 for 22 patients (46%). The median NIHSS score at discharge was 25 (range, 3–38). Seventeen patients (35.4%) died, and 17 surviving patients (35.4%) could not live independently. The clinical data are shown in Table 1. 

### 3.2. Initial Radiological Findings

The cerebellar hemorrhage was located in the left cerebellar hemisphere in 15 patients (31.2%), in the right cerebellar hemisphere in 19 patients (39.6%), and in the vermis in 14 patients (29.2%). The maximal diameter of the cerebellar hematomas in the 48 patients ranged from 30 to 65 mm. The mean maximum diameter of the cerebellar hematomas was 48 mm. All patients had ventricular dilatation. A tight posterior fossa was present in 28 patients (58.3%), a compression of the brain stem in 38 patients (79.2%), and intraventricular blood in 33 patients (68.75%).

### 3.3. Surgical Treatment and Postoperative Outcome

Forty-four patients (92%) were treated by suboccipital craniectomy for evacuation of their cerebellar hematomas. Only four patients underwent ventriculostomy alone. We performed nine cerebellar hematoma evacuations by suboccipital craniectomy only, and 35 by suboccipital craniectomy with a ventriculostomy. Twenty-one cerebellar hematomas were evacuated within 6 h, and 27 after more than 6 h. Two of the nine patients who only underwent suboccipital craniectomy did not survive, and 13 of 35 patients who underwent suboccipital craniectomy with a ventriculostomy did not survive. Postoperative hemorrhage occurred after six cerebellar hematoma evacuations, and three of these patients did not survive. Postoperative outcomes among the 48 patients at two years, determined by reference to the GOS scores, were as follows: 14 had good outcomes (29.2%) and 34 had poor outcomes (70.8%); 17 of the 34 patients with poor outcomes were classified according to their survival outcomes. Table 2 demonstrates that GCS scores on admission, pre-operative GCS scores, NIHSS scores at discharge, and hematoma volume were associated with the postoperative outcomes. These results are confirmed by a univariate analysis as shown in Table 3.

### 3.4. Analysis of Factors with an Impact on Clinical Outcome

Multiple logistic regression analyses demonstrated that the NIHSS score at discharge (*p* = 0.018) had a significant adverse influence on the postoperative outcome. The analysis of these variables revealed that an increase of one point in a patient’s NIHSS score at discharge increased the probability of a poor two-year postoperative outcome by 28.5% in patients with acute SHC undergoing neurological surgery (*p* = 0.018, OR = 1.285, 95% CI = 1.045–1.580, Table 4). Moreover, the NIHSS is independent of the prognostic role of postoperative mortality (*p* = 0.025, OR = 1.103, 95% CI = 1.012–1.202, Table 5).

## 4. Discussion

Our study demonstrated an association between a poor outcome on the GOS two years after surgical treatment of an SCH and the NIHSS scores at discharge. Specifically, an increase of one point in the NIHSS score at discharge will increase the probability of a poor 2-year postoperative outcome by 28.5% in patients with an acute SCH who undergo neurological surgery.

Acute space-occupying cerebellar hemorrhages may cause morbidity or death due to medullary compression from tonsillar herniation through the foramen magnum, due to midbrain compression by vermian herniation through the tentorial hiatus, or due to direct brain stem compression. The first reported surgical evacuation of a cerebellar hematoma was performed 110 years ago by Balance, but considerable debate remains concerning the recommendations for the surgical treatment of an acute cerebellar hematoma. Despite the various treatment strategies proposed by different authors, the latest American Heart Association and the American Stroke Association (AHA/ASA) guidelines recommend that patients with cerebellar hemorrhage who are deteriorating neurologically or who have brainstem compression and/or hydrocephalus from a ventricular obstruction should undergo surgical removal of the hemorrhage as soon as possible [6]. The determinants of operative management are usually the hematoma size and the neurological examination findings [12,13]. 

Both, hematoma sizes of 3 cm in diameter or larger, and neurological exam results worse than a GCS score of 13 have been used as determinants for operative intervention in many reports and studies [2,14,15,16]. The patients in our series all presented with a hematoma size of ≥3.0 cm. Some authors have recommended that patients with good neurological condition (GCS ≥ 13) undergo evacuation of their hematomas if the size is >3.0 cm or if significant compression of the 4th ventricle is present, in order to prevent potential deterioration [2,3,17,18]. Several nonrandomized studies have suggested that patients with cerebellar hemorrhages >3 cm in diameter, or patients whose cerebellar hemorrhages are associated with brainstem compression or hydrocephalus have better outcomes with surgical decompression [18,19,20]. In the present single-center analysis, 48 patients with an acute cerebellar hemorrhage were treated using a standardized surgical technique over a 5-year period. Most patients (39/48) underwent operations within 24 h of ictus. Postoperative rebleeding occurred in only six patients in our series, and the clinical long-term outcome was good in 29.2% of patients; nevertheless, the mortality remained high at 35.4%. 

Surgical complications may occur, and recurrent hemorrhage frequently results in a worse outcome [20,21]. A previous study reported that only 4 of 28 patients who were operated on (14%) had a favorable outcome at 49 months post-surgery, and another reported that 57 patients who were operated on showed a 25% mortality rate and only a 47% favorable outcome rate [5,22]. The overall clinical outcome of patients in the present study therefore falls within the range of the other large series of surgically treated patients with a spontaneous cerebellar hemorrhage [3,12,23]. Taken together, the results suggest that an acute cerebellar hemorrhage remains associated with high rates of mortality and morbidity, although the majority of patients who do survive subsequently experience a good outcome with the ability to function independently.

Poor outcomes in patients treated for an SCH have been associated with various radiological factors, including hematoma diameter of >3 cm, hydrocephalus, the presence of intraventricular hemorrhage, and the degree of compression of the quadrigeminal cistern or fourth ventricle [2,3,20,21,24,25,26]. For example, Dammann et al. described the presence of a tight posterior fossa and brain stem compression on the initial CT scan as significant predictive factors for a poor outcome [5]. Similarly, Weisberg et al. described a correlation between poor clinical outcome and the presence of tight posterior fossa (TPF) in a small series of 14 patients [9]. The study by St Louis et al. identified the following features on a CT scan as strong predictors of poor outcome: (a) hematoma of >3 cm in diameter; (b) brainstem compression; (c) hydrocephalus; and (d) intraventricular extension from the cerebellar hemorrhage [26]. Apart from the hematoma size, most studies found that the radiological findings of compression of the basal cisterns or compression of the 4th ventricle were predictive for poor clinical outcome, although a 4th ventricle compression was not a significant predictor in the present study. In addition, in our study, although the hematoma volume influenced the clinical outcome in the univariate analysis, the multivariate analysis did not identify any neuroradiological characteristics as independent prognostic factors.

Several clinical features in our study were predictive for a poor outcome, including sex, GCS on admission, pre-operative GCS, NIHSS at discharge, and hematoma volume. We did not find age, surgical timing, postoperative rebleeding, or hematoma diameter to be significant predictive factors for a poor outcome. Surgical timing and surgical method also did not significantly impact the outcome. Nevertheless, the significant correlations with a poor outcome suggested a harboring of covariates, so we further partitioned the data and extracted the most important discriminating factors. The NIHSS score at discharge significantly predicted poor outcome in patients with an SCH following neurological surgery. 

Our findings are confirmed by some previous studies that analyzed various predictors of clinical outcome. For example, Pollak et al. found a strong correlation between the initial neurological condition and clinical outcome [27]. The larger series of Da Pian, van Loon, Donauer, and Dolderer also found a significant correlation between the initial neurological condition and clinical outcome [18,20,22,28]. Similarly, some studies that used the NIHSS, which can assess acute ischemic stroke severity and outcome [29,30,31,32], have reported that higher NIHSS scores are associated with an increased severity of vascular lesions [32,33,34]. The present study is the first to analyze the predictive value of the NIHSS score at discharge for a poor outcome in patients with an SCH. 

One limitation of the present study was that the need for, and the timing of, surgical intervention was left to the discretion of the attending neurosurgeon. In other words, possible selection bias may raise some caveats in interpreting the outcome of the results. However, despite its retrospective nature and its relatively small sample size, our study provides a more nuanced understanding of the postoperative outcome in cases of cerebellar hemorrhage and the findings are helpful when framing prognostic estimates for family members. Moreover, the widely distributed range of patient ages indicated the confounding factors of different etiologies related to cerebellar hemorrhage. The data presented here can be used to predict the two-year postoperative outcome of a cerebellar hemorrhage and to plan for an aggressive or supportive course of management.

In conclusion, the results of the present study demonstrated that 70.83% of patients with acute cerebellar hemorrhage had poor postoperative outcomes, namely death, vegetative state, or severe disability. Our study also revealed a significant association between the NIHSS scores at discharge and the postoperative outcomes in patients with primary cerebellar hemorrhage. An increase of one point in a patient’s NIHSS score at discharge could increase the probability of a poor two-year postoperative outcome by 28.5% in patients with an acute SCH who undergo neurological surgery. 

## 5. Conclusions

An increase of one point in a patient’s NIHSS score at discharge increased the probability of a poor two-year postoperative prognosis by 28.5% in patients with acute SHC undergoing neurological surgery. Moreover, the NIHSS is an independent prognostic marker of postoperative mortality. These results concluded that an increased NIHSS score at discharge is associated with a poor two-year postoperative outcome in patients with spontaneous cerebellar hemorrhage who undergo neurological surgery.

## Figures and Tables

**Table 1:** Baseline characteristics of patients with spontaneous cerebellar hemorrhage (SCH).

	Glasgow Outcome Scale (2 years)		*p* Value
	Good (*n* = 14)	Poor (*n* = 34)	
Sex			0.045
Male	11 (78.6)	16 (47.1)	
Female	3 (21.4)	18 (52.9)	
Surgical timing			0.575
≤6 h	7 (50.0)	14 (41.2)	
>6 h	7 (50.0)	20 (58.8)	
Operative method			0.944
Suboccipital craniectomy only	3 (21.4)	6 (17.6)	
Ventriculostomy only	1 (7.1)	3 (8.8)	
Suboccipital craniectomy and ventriculostomy	10 (71.4)	25 (73.5)	
Postoperative rebleeding			0.656
No	13 (92.9)	29 (85.3)	
Yes	1 (7.1)	5 (14.7)	
Alcohol			1.000
No	12 (85.7)	30 (88.2)	
Yes	2 (14.3)	4 (11.8)	
Smoking			0.339
No	11 (78.6)	31 (91.2)	
Yes	3 (21.4)	3 (8.8)	
Diabetes mellitus			0.656
No	13 (92.9)	29 (85.3)	
Yes	1 (7.1)	5 (14.7)	

**Table 2:** Clinical factors influencing postoperative outcome of patients with spontaneous cerebellar hemorrhage (SCH).

	GOS at 2 Years		*p* Value
	Good (*n* = 14)	Poor (*n* = 34)	
Age	61.1 ± 11.4 (63, 43–77)	63.8 ± 9.6 (63, 48–84)	0.401
GCS on admission	11.9 ± 3.0 (13, 6–15)	7.5 ± 4.6 (6, 3–15)	0.002
Pre-operative GCS	10.5 ± 2.9 (9, 6–14)	5.9 ± 3.6 (4, 3–15)	<0.001
NIHSS at discharge	10.6 ± 4.8 (12, 3–19)	30.4 ± 9.9 (36, 7–38)	<0.001
Hematoma volume	16.6 ± 5.2 (16, 8–27)	23.2 ± 7.9 (22, 14–39)	0.006
Hematoma diameter	46.5 ± 9.0 (46, 31–63)	48.6 ± 7.9 (47, 30–65)	0.426

Data are mean ± SD (median, range). *p* values are based on a Student’s *t*-test or Mann-Whitney U test. Abbreviations: GCS = Glasgow Coma Scale; GOS = Glasgow Outcome Scale; NIHSS = National Institutes of Health Stroke Scale.

**Table 3:** Univariate analysis for predictors of postoperative outcome in patients with spontaneous cerebellar hemorrhage (SCH).

	Poor Outcome	
	Odds Ratio (95% CI)	*p* Value
Age	1.028 (0.965–1.096)	0.393
Sex (female)	0.242 (0.057–1.027)	0.054
GCS on admission	0.790 (0.669–0.933)	0.006
Pre-operative GCS	0.721 (0.590–0.880)	0.001
NIHSS at discharge	1.310 (1.086–1.580)	0.005
Hematoma volume	1.165 (1.032–1.316)	0.014

Abbreviations: GCS = Glasgow Coma Scale; NIHSS = National Institutes of Health Stroke Scale.

**Table 4:** Multivariate analysis for independent predictors of postoperative outcome in patients with an SCH.

	Poor Outcome	
	Odds Ratio (95% CI)	*p* Value
Age	0.988 (0.862–1.132)	0.862
Sex (female)	0.758 (0.063–9.197)	0.828
GCS on admission	1.068 (0.648–1.760)	0.795
Pre-operative GCS	0.687 (0.357–1.323)	0.261
NIHSS at discharge	1.285 (1.045–1.580)	0.018
Hematoma volume	0.966 (0.768–1.215)	0.767

Abbreviations: GCS = Glasgow Coma Scale; NIHSS = National Institutes of Health Stroke Scale.

**Table 5:** Multivariate analysis for independent predictors of postoperative mortality in patients with an SCH.

	Poor Outcome-Postoperative Mortality	
	Odds Ratio (95% CI)	*p* Value
Age	1.039 (0.944–1.145)	0.433
Sex (female)	1.539 (0.302–7.838)	0.603
GCS on admission	0.989 (0.766–1.277)	0.932
Pre-operative GCS	0.815 (0.561–1.183)	0.281
NIHSS at discharge	1.103 (1.012–1.202)	0.025
Hematoma volume	0.983 (0.879–1.099)	0.757

Abbreviations: GCS = Glasgow Coma Scale; NIHSS = National Institutes of Health Stroke Scale.

## Data Availability

The data that support the findings of this study are available on request from the first author, Lee TH.

## Abbreviations

AHA/ASA	American Heart Association and the American Stroke Association;
CT	Computed tomography;
EVD	External ventricular drainage;
ICH	Intracerebral hemorrhage;
GOS	Glasgow outcome scale;
GCS	Glasgow Coma Scale;
NIHSS	National Institutes of Health Stroke Scale;
SCH	Spontaneous cerebellar hemorrhage;
SD	Standard deviation
